# Minimally invasive transvaginal single-port laparoscopic vesicovaginal fistula repair: a case report and the point of this technique

**DOI:** 10.3389/fsurg.2024.1331476

**Published:** 2024-01-19

**Authors:** Jianbiao Huang, Yu Cheng, Bin Wang, Haichao Chao, Xiangda Xu, Tao Zeng

**Affiliations:** The Second Affiliated Hospital, Jiangxi Medical College, Nanchang University, Nanchang, Jiangxi, China

**Keywords:** transvaginal, single-port laparoscopy, vesicovaginal fistula, repair, case report

## Abstract

The optimal surgical method of vesicovaginal fistula (VVF) remains uncertain. Minimally invasive surgical approaches have become highly popular in line with technological advancements, namely, laparoscopic, robotic, and transvaginal techniques. However, these techniques still require invasiveness. This is the first case report that described a novel “zero-incision” technique for natural orifice transvaginal single-port laparoscopy used to repair a recurrent and high-position VVF. The patient underwent transvaginal single-port laparoscopic repair of a VVF. Methylene blue was used to locate the VVF, and a needle electrode was used to thoroughly remove the scar tissue of the VVF. In addition, this technique for transvaginal single-port laparoscopy provides more working space to expose and repair fistulas conveniently and adequately. One year after surgery, the patient remained asymptomatic and had no fistula recurrence. Minimally invasive transvaginal single-port laparoscopy provides a clear surgical field, is safe and feasible. This novel technique has promising as an additional personalized treatment option for VVF repair.

## Introduction

A vesicovaginal fistula (VVF) is an abnormal anatomical connection between the vagina and bladder that causes continuous and involuntary urinary leakage from the vagina and has a serious impact on patients' quality of life. The etiology of VVF varies, and the socioeconomic status of the country affects its incidence. Obstetric trauma resulting in a VVF is more commonly found in developing countries, whereas iatrogenic injuries during gynecological surgeries, such as hysterectomy, are the primary causes in developed countries.

Management options for VVF include both conservative and surgical approaches, although there is currently no consensus on the optimal treatment. The choice of treatment depends on the disease characteristics and the surgeon's preference. Conservative treatments may be considered the initial approach for small (<10 mm), clean, nonmalignant fistulas, with a reported success rate of 5%–11% (1).

Numerous surgical techniques have been described for repairing VVFs, such as laparotomy, transvaginal laparoscopy, robot-assisted surgery, and other minimally invasive techniques. Minimally invasive approaches, including a hybrid technique utilizing cystoscopy and intravesical treatment, have shown promising outcomes (2). Nonetheless, these techniques still involve some degree of invasiveness. In this report, we present a case of iatrogenic VVF that was repaired via a novel “zero-incision” technique called natural orifice transvaginal endoscopic (NOTE). This approach involves advancing a single-port laparoscopic trocar through the vagina, which is a natural orifice, to repair the fistula tract. This technique is potentially less invasive for VVF repair.

## Case presentation

A 53-year-old female patient presented to our hospital complaining of continuous urine leakage from the vagina as a result of an abdominal hysterectomy that was performed at a local hospital due to cervical cancer (the patients did not undergo a radiotherapy) two years prior. Urine leakage from the vagina occurred in perioperative period and the VVF was diagnosed at that time, the patient needed two pads one day. Half a year later, she underwent an open abdominal transvesical repair of the fistula (the VVF was located at the apex of the vagina, a conventional transvaginal repair would likely fail) at another medical center. However, urinary leakage persisted following surgery and the patient still need one pad one day. There were no other chronic comorbidities for the patient. Due to the high location of the VVF, it could be challenging to adequately expose and suture the fistula correctly through a conventional transvaginal approach. Attempting a transabdominal or laparoscopic approach would also be problematic due to extensive adhesions and anatomical distortion. Considering these challenges, a novel technique called transvaginal single-port laparoscopic VVF repair was deemed appropriate. The patient was fully informed about this new approach and provided written consent for the surgery and potential publication of the case.

## Surgical procedure

**Figure 1:**
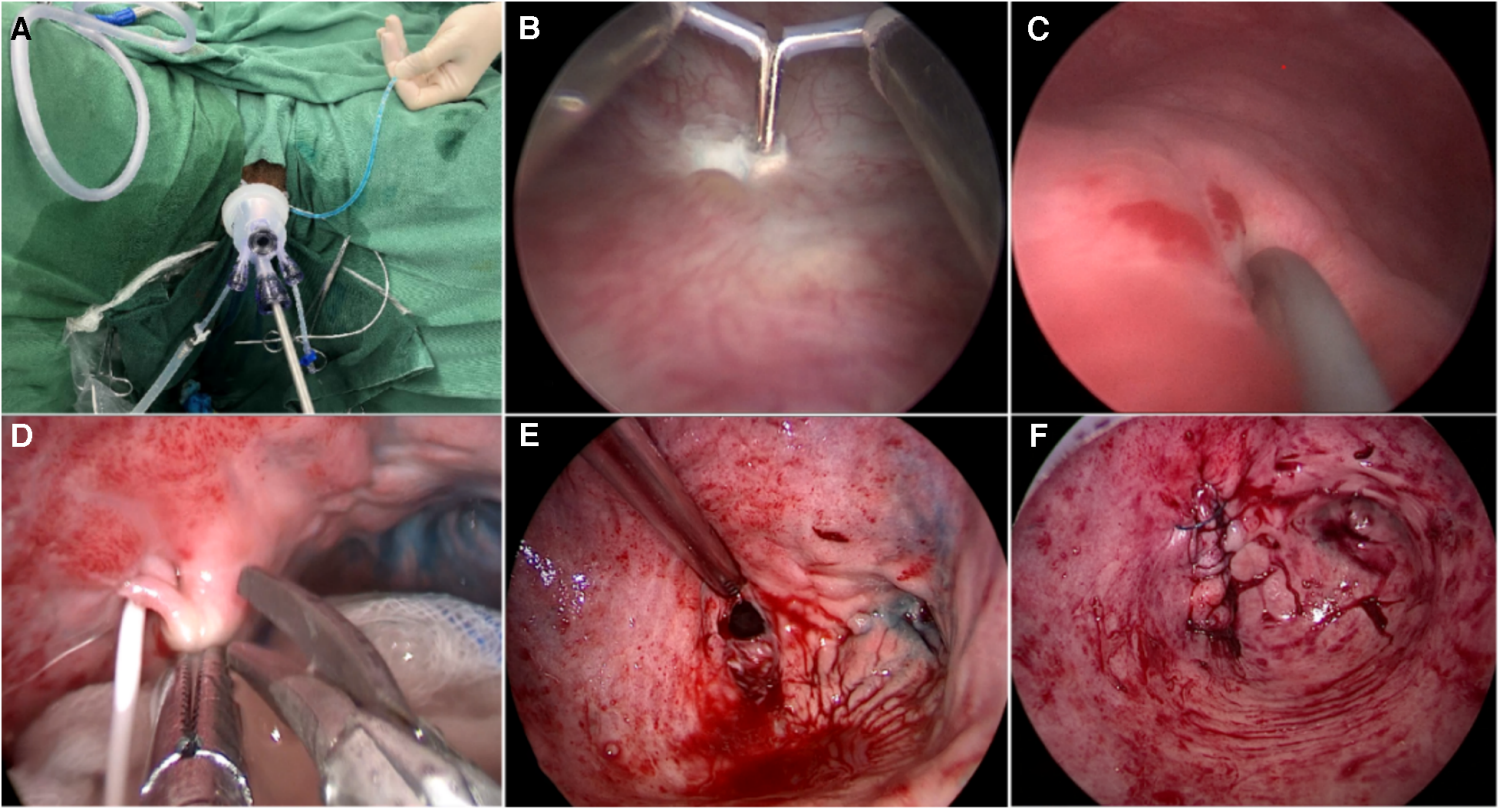
Surgical procedure of transvaginal single-port laparoscopic vesicovaginal fistula repair. (**A**) A single-port laparoscope was introduced into the vagina, and the vagina was expanded by insufflation with CO2 at a pressure of 6–8 mmHg. (**B**) A needle electrode was used to make a 0.5 cm circumferential incision to thoroughly remove scar tissue in the bladder around the fistula. (**C**) A ureteric catheter was passed under vision through the VVF from the bladder to the vagina. (**D**) Excision of 1-cm of the mucosa and muscle surrounding the vaginal scar tissue and the fistula with scissors. (**E**) The VVF tract was similar to a trumpet. (**F**) Successive single-layer closure of vesical and vaginal fistulas with 3-0 V-Loc barbed sutures under transvaginal single-port laparoscopic vision.

**Figure 2:**
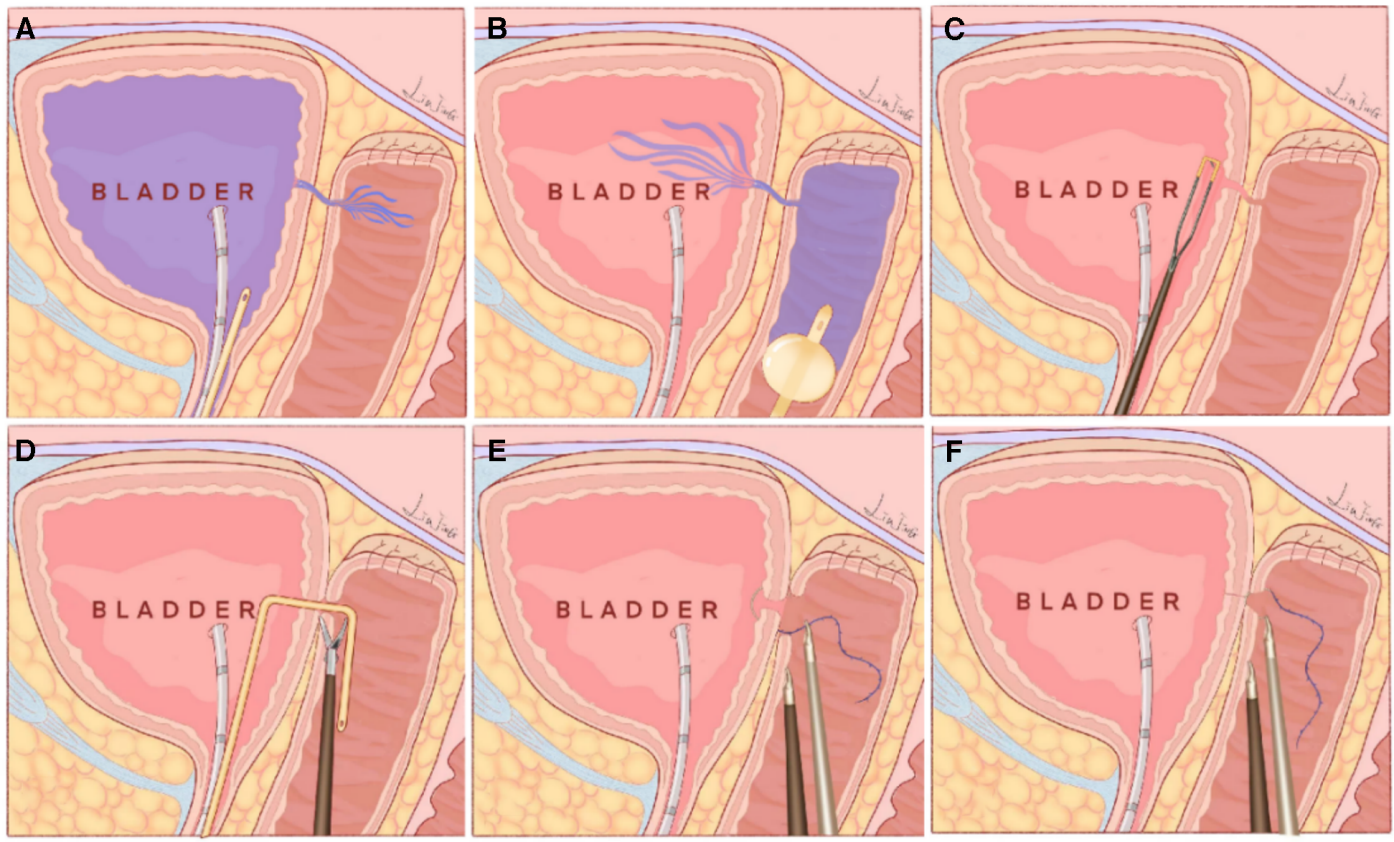
Schematic diagram of transvaginal single-port laparoscopic vesicovaginal fistula repair, which shows the innovations of this technique. (**A**) A 20 F Foley catheter was inserted into the bladder, 200 ml methylene blue solution was injected and immediately gushed from the apex of the vagina, thereby showing the fistula opening in the vagina. (**B**) A 20 F Foley catheter was inserted into the vagina, and the balloon was filled with 60 ml of saline solution. Then, 150 ml of methylene blue solution was injected into the vagina, and the cystoscope showed methylene blue gushing from the fistula opening in the bladder. (**C**) A needle electrode was used to make a circumferential incision to thoroughly remove scar tissue in the bladder around the fistula. (**D**) A ureteric catheter was passed under vision through the VVF from the bladder to the vagina. The single-port laparoscope was introduced into the vagina again. The scar tissue surrounding the fistula in the vagina was removed using scissors. (**E**) The fistula opening in the bladder was closed using 3-0 V-Loc barbed suture under transvaginal single-port laparoscopic vision. (**F**) The fistula opening in the vagina was sutured using a 3-0 V-Loc barbed suture under transvaginal single-port laparoscopic vision.

After general anesthesia, the patient was placed in the lithotomy position. A 22 F cystoscope was used to begin the procedure in the bladder. The bilateral ureteral orifices were seen clearly, and a 6 F ureteric catheter was inserted into the ureter. However, the fistula opening was not found. A single-port laparoscope was introduced into the vagina, and the vagina was expanded by insufflation with CO2 at 6–8 mmHg pressure (Figure 1A). The laparoscope showed the closed apical vagina but no fistula. Methylene blue solution that was injected into the bladder through a Foley catheter immediately gushed from the apex of the vagina, thereby revealing a 4 mm fistula. After removal of the single-port laparoscope, a 20 F Foley catheter was inserted into the vagina, and the balloon was filled with 60 ml of saline solution. Gauze soaked with iodophor was placed in the vagina. Methylene blue solution was injected through the Foley catheter, and the cystoscope showed methylene blue gushing from the right side of the bladder trigone region, the fistula was 3 cm away from the right ureteral orifice. Then, the scar tissue around the fistula was removed by making a 0.5-cm incision using a needle electrode (Figure 1B). A ureteric catheter was passed, under direct vision, from the bladder to the vagina through the VVF (Figure 1C). The single-port laparoscope was reintroduced into the vagina to allow mucosal and muscular excision of 1-cm vaginal scar tissues surrounding the fistula using scissors (Figure 1D). The fistula tract was shaped like a trumpet (Figure 1E). Finally, single-layer closure of vesical and vaginal fistulas was performed using 3–0 V-Loc barbed sutures under transvaginal single-port laparoscopic guidance (Figure 1F). We injected diluted methylene blue solution to check for fluid leakage from the anterior wall of the vagina. The vagina was filled with iodophor yarn strips, and the surgery was completed, the whole operation took two hours. There was minimal bleeding and no intra or postoperative complications. The patient was catheterized for two weeks and maintained antibiotic therapy for six days and hospitalized for six days. Figure 2 illustrates the schematic diagram of transvaginal single-port laparoscopic vesicovaginal fistula repair and highlights the key points of the technique. The patient remained asymptomatic with no recurrence of the VVF after a half year.

## Discussion

Vesicovaginal fistula is the most common type of genitourinary fistula in females and can have significant physical and psychological impacts. Diagnosing and treating VVF requires caution due to its iatrogenic causes, and a definitive diagnosis typically relies on standardized and convincing evidence. Imaging methods such as intravenous pyelography or computed tomography urography can confirm the diagnosis. Cystoscopy after methylene blue injection can provide further information about the location, size, and number of fistulas. When planning the treatment for VVF, meticulous care is necessary, especially when considering surgical intervention. A well-designed surgical plan is crucial to ensure the success of the operation, as the local conditions at the operative site are often most favorable during the initial repair, thus maximizing the chances of a successful outcome. If the first repair fails, subsequent treatment attempts may become more challenging. However, there is currently no standardized protocol determining the optimal surgical route and timing for VVF treatment. The choice of surgical approach depends on various factors, including the location, size, number of fistulas, vaginal conditions, surgeon's expertise, and patient preferences.

The traditional transvaginal approach is the most commonly used surgical method for repairing VVF. It has several advantages, including a shorter surgical time, less intraoperative bleeding, a shorter hospital stay, faster postoperative recovery, and high success rates. It is a minimally invasive procedure that can be repeated if needed, regardless of the timing of recurrence or repeat repair (3). However, there are some limitations to this approach. The transvaginal technique may be associated with an increased risk of vaginal shortening. It can be challenging to adequately expose and repair high-position VVFs, as well as complex and recurrent VVFs. Suturing these fistulas correctly can also pose difficulties. In such cases, a conventional transabdominal repair is often recommended (4). The transabdominal approach creates more space for meticulous preparation of the bladder and vaginal wall. It facilitates the identification of scar tissue and fistulas, thereby allowing for complete excision of inflamed tissues and ensuring proper mobilization of the bladder wall. The abdominal approach creates a secure foundation for tension-free closure of the bladder. Although studies have reported similar success rates between transabdominal and transvaginal surgeries, the former is more invasive and requires a longer hospital stay. Additionally, it has a higher risk of complications (4).

In recent years, minimally invasive techniques such as laparoscopic and robotic repair, were first reported in 1994 and 2005, respectively (5, 6), and have emerged as promising techniques for the management of VVFs because of their safety, feasibility, and effectiveness in various studies. Compared to open surgery, laparoscopic repair is associated with a lower morbidity rate and comparable success rates (7). A systematic review conducted by Miklos et al. focused on laparoscopic and robot-assisted VVF repair (7). The review demonstrated an overall success rate of 80%–100% for laparoscopic repair, with follow-up periods ranging from 1 to 74 months. The results suggest that laparoscopic repair is a reliable and successful treatment option for VVFs. Robot-assisted repair is particularly promising for high supratrigonal fistulas. It offers optimal exposure to the fistula area, allowing for wide excision of the fistula tissue. Robot-assisted repair has shown good success rates and lower morbidity rates in these cases. However, it is important to note that laparoscopic repair can be challenging due to the tricky preparation of previously damaged tissue and the suturing process. These difficulties need to be carefully addressed to ensure successful outcomes. Additionally, the robotic approach is associated with higher costs, which may limit its accessibility in some health care settings.

The laparoscopic approach, despite its advantages, is still considered invasive due to the requirement for several incisions. Studies have shown that nearly three-quarters of VVFs can be repaired vaginally, with a success rate that is comparable to that of transabdominal path repair and no significant differences (8). Transvaginal repair offers several benefits, including being more cost-effective than transabdominal repair. Thus, transvaginal repair is currently becoming increasingly valued and favored (3). A novel technique in which the advantages of both laparoscopic and transvaginal approaches were combined has been developed. This technique, a minimally invasive operation, involves the insertion of a single-port laparoscope through the vagina and thus allows adequate exposure of the VVF. This innovative approach incorporates the principles of natural orifice transluminal endoscopic surgery, which was first proposed by Mack in 2001 and has been applied in various urologic and gynecologic procedures (9). Galan et al. demonstrated the effectiveness and benefits of natural orifice transurethral endoscopic VVF treatment through their own case reports (10). It is believed that the transvaginal endoscopic method is superior to the transurethral route for treating VVFs, maybe especially for complex and recurrent cases. This technique has several advantages. First, use of a Foley catheter to find the fistula opening in the bladder (11): by using a Foley catheter, the fistula opening in the bladder can be precisely located, thus aiding in the identification and treatment of the VVF. Second, scar tissue removal with a needle electrode: the transvaginal endoscopic method with a needle electrode allows thorough removal of scar tissue in the bladder, ensuring optimal closure and healing of the VVF. Third, a larger working space and more convenience: this technique creates more room for maneuverability and better access to adequately expose the fistula, remove scar tissue in the vagina, and suture the fistula in layers using a transvaginal single-port laparoscope. Other advantages of the natural orifice transvaginal endoscopic technique include no incision and better visualization. Overall, the transvaginal single-port laparoscopic approach has the potential as an additional personalized treatment option for selected VVF repair.

## Strengths and limitations

The transvaginal single-port laparoscopic technique proposed in this study effectively meets the requirements for successful VVF repair. It provides adequate exposure, good anatomical assessment, allows precise dissection, tension-free suturing, proper postoperative bladder drainage, and provides sufficient blood supply for tissue healing. This technique is valuable in VVF treatment because of its ability to address these crucial aspects. To the best of our knowledge, this study includes the first reported transvaginal single-port laparoscopic repair of VVF and highlights the key advantages of this technique. These include the use of a Foley catheter to locate the fistula opening in the bladder, thorough removal of scar tissue using a needle electrode, and establishment of a larger working space for convenient and optimal exposure, scar tissue removal, and suturing using a transvaginal single-port laparoscope. However, additional multicenter studies with larger patient populations are needed to evaluate the effectiveness of this technique and to establish recommendations for its use.

## Conclusion

In conclusion, minimally invasive transvaginal single-port laparoscopic repair of VVFs has several advantages. The procedure has shown promising outcomes and is considered a safe option for VVF repair.

## Data Availability

The raw data supporting the conclusions of this article will be made available by the authors, without undue reservation.
